# Are social norms associated with smoking in French university students? A survey report on smoking correlates

**DOI:** 10.1186/1747-597X-4-4

**Published:** 2009-04-02

**Authors:** Lionel Riou França, Bertrand Dautzenberg, Bruno Falissard, Michel Reynaud

**Affiliations:** 1 INSERM U669, Maison de Solenn 97, bvd de Port-Royal, 75679, Paris, Cedex 14, France; 2 Université Paris 6, Pierre et Marie Curie, 4 place Jussieu, 75005, Paris, France; 3 Faculté de Médecine Pierre et Marie Curie, UPRES EA2397, 91, 105 boulevard de l'Hôpital, 75013 Paris, France; 4 Assistance Publique-Hôpitaux de Paris (AP-HP), Service de pneumologie et réanimation, GH Pitié Salpêtrière Division Montyon, 75651, Paris, cedex 13, France; 5 Université Paris-Sud 11 and Université Paris Descartes 5, UMR-S0669, Maison de Solenn, 97, boulevard de Port-Royal, 75679 Paris cedex 14, France; 6 Assistance Publique-Hôpitaux de Paris (AP-HP), Hôpital Paul Brousse, département de santé publique 12, avenue Paul-Vaillant-Couturier, 94804, Paris, Villejuif Cedex, France; 7 Assistance Publique-Hôpitaux de Paris (AP-HP), Hôpital Paul Brousse, unité fonctionnelle d'addictologie, 12, avenue Paul-Vaillant-Couturier, 94804, Paris, Villejuif Cedex, France

## Abstract

**Background:**

Knowledge of the correlates of smoking is a first step to successful prevention interventions. The social norms theory hypothesises that students' smoking behaviour is linked to their perception of norms for use of tobacco. This study was designed to test the theory that smoking is associated with perceived norms, controlling for other correlates of smoking.

**Methods:**

In a pencil-and-paper questionnaire, 721 second-year students in sociology, medicine, foreign language or nursing studies estimated the number of cigarettes usually smoked in a month. 31 additional covariates were included as potential predictors of tobacco use. Multiple imputation was used to deal with missing values among covariates. The strength of the association of each variable with tobacco use was quantified by the inclusion frequencies of the variable in 1000 bootstrap sample backward selections. Being a smoker and the number of cigarettes smoked by smokers were modelled separately.

**Results:**

We retain 8 variables to predict the risk of smoking and 6 to predict the quantities smoked by smokers. The risk of being a smoker is increased by cannabis use, binge drinking, being unsupportive of smoke-free universities, perceived friends' approval of regular smoking, positive perceptions about tobacco, a high perceived prevalence of smoking among friends, reporting not being disturbed by people smoking in the university, and being female. The quantity of cigarettes smoked by smokers is greater for smokers reporting never being disturbed by smoke in the university, unsupportive of smoke-free universities, perceiving that their friends approve of regular smoking, having more negative beliefs about the tobacco industry, being sociology students and being among the older students.

**Conclusion:**

Other substance use, injunctive norms (friends' approval) and descriptive norms (friends' smoking prevalence) are associated with tobacco use.

University-based prevention campaigns should take multiple substance use into account and focus on the norms most likely to have an impact on student smoking.

## Background

The social norms theory claims that individuals align many of their health and social behaviours with their beliefs concerning the prevalence and acceptability of these behaviours in their environment. Accordingly, overestimations of unhealthy behaviours will increase these behaviours, and underestimations of healthy behaviours will discourage individuals from engaging in them [1]. Peer influences are therefore of interest in understanding tobacco use. Perceived prevalence of smoking by peers appears to be associated with individual smoking in several studies among U.S. students from grade 7 (12–13 years) to grade 12 [2-6], although not all studies are consistent. One study finds, when adjusting for other factors, that perceived prevalence of teenage smoking tended to be lower among past-week and daily smokers (8 to 10^th ^grade students) [7]. Another finds that the risk of being a smoker two years later increases with the perception of the prevalence of peer smoking for 7^th ^graders, but decreases for 9^th ^graders [2]. Other norms for use have also been identified, such as perceived approval of smoking [8].

Most of the research published focuses on high school students, even though tobacco initiation is not uncommon among higher education students [9].

Adapted and effective prevention strategies for students suppose that we have knowledge of the factors associated with smoking in this population. Furthermore, there is a need to tailor the messages, since students might not feel concerned by general population prevention campaigns [10]. As students tend to overestimate the actual proportion of peers who are smokers [11], prevention campaigns providing more accurate norms with respect to current prevalence and acceptability of tobacco use have the potential to reduce or inhibit smoking.

In his review of research on social norms, Berkowitz states that peer influences "have a greater impact on individual behaviour than biological, personality, familial, religious, cultural and other influences" [1]. While most of the references he cites to back up this claim are based on alcohol use research, other studies show that normative beliefs are also influential in smoking initiation [8]. There are, however, many other variables that have been reported to be associated with smoking. Among higher education students, these include gender (some studies finding that men are more at risk of tobacco use, experimentation or addiction than women [12-15], others finding the opposite [16,17] and some finding no association [18,19]), age [16], alcohol use [9,12,20-27] including heavy episodic drinking [16], cannabis use [16,20,23-25,28], body mass index (BMI) among women [28] and academic discipline [29-31]. At university level, some policies are influential: preventive education related to smoking and smoking cessation courses decrease the likelihood of smoking, while having designated smoking areas increases it [31]. On a related note, being supportive of smoke-free universities is less common among smokers [32,33]. Finally, tobacco-related perceptions, such as smoking outcome expectancies [34] or believing that smoking experimentation is safe [35], are also linked to smoking.

Other factors of potential interest for prevention purposes have been identified in other populations. Among adults, living with a partner was found to be associated with success in quitting [36]. In his evaluation of "counterindustry" media campaigns, Hersey et al showed that attitudes towards and beliefs about the tobacco industry are associated with smoking intentions and behaviour among 12–24 year-olds [37]. School policies about smoking and their enforcement were found to be associated with smoking [38,39]. Self-esteem was found to be associated with smoking among boys aged 13–16 [40]. Family structure was associated with smoking in a sample of 14–16 year-old pupils [41]. Low academic achievement has been documented to be a predictor of smoking among 10–18 year-old adolescents [42].

In France, in a population of 12–26 year-olds, smoking prevalence was the highest among 19–21 year-olds [43]. Higher education students are less often smokers than non-students (43% vs. 49%, p < 0.05) and they smoke less (average number of cigarettes smoked among smokers of 9.4 vs. 12.2 per day) [44]. However, university students are easier to target, which makes prevention campaigns in universities worthwhile. Furthermore, there is some evidence that peers with higher educational status are more influential on smoking cessation [45] and higher education students could therefore be more receptive to social norms.

The objectives of this study were to characterise smoking behaviours and their correlates among selected French university students and to test the hypothesis of an association between smoking and social norms. Findings from this study will be used to develop a prevention campaign based on social norms.

Social norms being only one part of the identified risk factors for smoking, they are only interesting for prevention purposes if they are found to be significantly associated with smoking even after controlling for other influential risk factors. The existence of such an association needs therefore to be assessed keeping in mind that other predictors of smoking do exist. The first contribution of this study to existing research is that we assess the impact of social norms in relation to a large number of other identified risk factors for smoking. The second contribution derives from the focus on an older sample of students. While the factors associated with smoking among young adolescents have been extensively described, less is known about university students.

The third contribution resides in the population studied, which originates from France. Most of the literature specific to tobacco smoking epidemiology among higher education students originates from U.S. universities. Of the 113 references retrieved by a MEDLINE bibliographic search from January 1995 to June 2008, 68 are from the U.S. and only 10 from Europe (counting Turkey as a European country). In the only published study reporting results for France, cigarette smoking prevalence, in 2000, was 31.5% for men and 34.8% for women [46]. These figures are slightly higher than those reported in 1999 for U.S. students (28.4% for men and 28.5% for women) [25].

## Methods

### Data

The REACTIF study (*Rendu Enquête Alcool Cannabis Tabac en Île de France*, the report on the *Ile de France *study about alcohol, cannabis and tobacco results) surveys university students in their second and third year of studies. The study was approved by the institutional review board of the French institute of health. The results presented here are those of the first survey in the REACTIF study. The study was limited to 4 academic disciplines (medicine, sociology, nursing and English as a foreign language) in order to minimise variability among the classes compared.

The choice of focusing on second-year university students was motivated by the selection process occurring among first-year students in France: at the end of their first year, many change their courses, give up their studies, or simply repeat their year. Two cross-sectional surveys in the second and third years were expected to yield a more stable population.

The choice of nursing studies was motivated by the particularly large prevalence of tobacco use in this population in France [47]. Sociology students were also expected to have a high smoking prevalence [30]. In contrast, medicine and foreign language students were expected to have a lower prevalence of smoking.

The sampling unit was the class, with the constraint of having only one class per faculty. Faculties were selected at random in the *Ile de France *region (around Paris). The dean of the university was to give consent to the study for the faculty to be selected. We included 4 nursing schools, 3 sociology faculties, 3 foreign language faculties and 2 medicine faculties. All second-year students within the selected programs were included in the study.

The first survey took place between October 2005 and February 2006. The 13-page, anonymous and voluntary paper-and-pencil questionnaire was completed by 731 second-year university students during a specified time frame at a scheduled lecture. Of students attending the designated lectures, 98% participated in the survey (approximately ten refused to complete the survey, mostly due to scheduling).

Universities were unable to provide the exact number of students enrolled so that real lecture attendance rates are impossible to calculate.

### Smoking status

Three key questions defined smokers. First, the students were asked how many days they had smoked *in the 30 days preceding the survey*. Second, the students reporting having smoked in the last 30 days were asked how many days per 30-day month they *usually *smoked tobacco. Third, they were asked how many cigarettes they usually smoked per day on the days when they did smoke.

The dependent variable considered is the number of cigarettes (hand rolled or in packs) usually smoked per month. It was obtained by multiplying smoking frequency by the number of cigarettes smoked per smoking day. A student was considered as a smoker if the number of cigarettes usually smoked per month was non-null.

For descriptive purposes exclusively, we further categorized smokers on the basis of the definitions of the Centers for Disease Control and Prevention [48]. Current smokers could be daily smokers (students who reported having smoked every day in the last 30 days) or occasional smokers (students having smoked, but not every day). Among students not currently smoking, former smokers had smoked at least 100 cigarettes in their lifetime and non-smokers had smoked less than 100 cigarettes.

### Independent variables

The primary independent variables of interest for the purposes of this study, which aims to identify the effect of social norms on cigarette smoking, are the social norms measures themselves. These norms can be classified as injunctive (inferences about other people's approval of smoking) or descriptive (perceptions of other people's behaviours) [1]. Tobacco-related social norms were assessed by way of 7 variables, measuring injunctive social norms (perceived approval by friends of: (a) smoking experimentation, (b) smoking occasionally, (c) smoking regularly), descriptive social norms (perceived prevalence of smoking (d) among peers from the same university in the academic discipline and (e) among friends), and environmental variables – (f) previous exposure to substance-use questionnaires and (g) perceived exposure to tobacco prevention campaigns.

To assess the relative importance of social norms for cigarette smoking, other smoking predictors were included. These were selected with three constraints. First, the variables were to be identified in the literature as potential smoking risk factors. Second, they were to enable comparisons with other French substance use surveys, such as the ESCAPAD survey (a substance-use survey on a representative sample of French youngsters – mostly 17 year-olds) [49] or the ESPAD survey (the European School Survey Project on Alcohol and other Drugs, based on 16 year-old students) [50]. Third, as our survey was not specific to tobacco use, but also described alcohol and cannabis use, we retained variables that could be measured for these three substances.

Overall, 24 additional variables were collected, describing student characteristics, the university environment, and tobacco perception. The 31 variables used in the analysis are presented in Table 1.

**Table 1:** The independent variables used in the model

**Variable**	**Description**
*Student characteristics*	

Gender	Male or female.
Age	Age ranged from 18 to 64 years.
Cannabis use	No use, use ≤ 1/week, use > 1/week in the previous year.
Alcohol use	No use, use < 10 days, use ≥ 10 days in the previous month.
Binge drinking	No episode, < 4 times, ≥ 4 times in the previous month.
Partner status	Being alone or having a partner.
Family structure	Parents living together or not.
Baccalauréat grade	Secondary school final exam grade, as a measure of social achievement.
Number of friends	0–4, 5–7, 8–10 or ≥ 11.
Number of friends in the class	0–1, 2–3, 4–5 or ≥ 6.
Self esteem	Rosenberg's self esteem scale.
BMI	Body mass index.

*University environment*	

Academic discipline	Sociology, medicine, English as a foreign language or nursing studies.
Smoking prevalence in the class	
Cannabis use prevalence	Estimated from the students' answers.
Drinking prevalence	
Binge drinking prevalence	
Smoke disturbance in university	"Are you disturbed by smoking in your university?": not at all, rarely, sometimes or often.
Smoke-free university support	"What is your position about your university being smoke-free?": completely positive, mostly positive, indifferent, somewhat against or completely against it.
Knowledge of university's tobacco policy	"Does your campus have a policy (e.g. consumption ban, ...) against tobacco?": no, yes or don't know.

*Social norms*	

Previous exposure to substance use questionnaires	Yes or no.
Perceived exposure to tobacco prevention campaigns	0 times, 1–3 times, 4–5 times, 6–30 times in the previous month.
Perceived prevalence of tobacco use among university peers	"Among 10 students, how many use tobacco?"
Perceived approval of tobacco experimentation by friends	"What would your close friends think if you tried to smoke tobacco once or twice?": wouldn't disapprove, would disapprove or would strongly disapprove.
Perceived approval of tobacco occasional use by friends	"What would your close friends think if you smoked tobacco occasionally?": wouldn't disapprove, would disapprove or would strongly disapprove.
Perceived approval of tobacco regular use by friends	"What would your close friends think if you smoked tobacco regularly?": wouldn't disapprove, would disapprove or would strongly disapprove.
Perceived prevalence of tobacco use among friends	"Among your friends, how many smoke tobacco?": none, less than one third, about half or more than two thirds.

*Measures related to tobacco*	

Tobacco perception score	"Do you think the following qualifiers are relevant to tobacco?" ("harmful", "a trap", "a pleasure", "healthy", "a scourge", "friendly and sociable") 6-item Likert scale from 0="Not at all" to 4="Entirely".
Attitudes towards the tobacco industry	Derived from a tobacco industry scale. 6-item Likert scale. Example: "Cigarette companies should have the right to sell".
Beliefs about the tobacco industry	Derived from a tobacco industry scale. 7-item Likert scale. Example: "Cigarette companies lie".
Tobacco prevention campaigns perception score	6-item Likert scale about tobacco prevention campaigns: "There are too many", "They are convincing", "I don't feel concerned", "They do not give the right reasons to change behaviour", "They catch attention", "They have more to do with political issues than with public health issues".

The *Baccalauréat *corresponds to the secondary school final examination, it is used as a measure of academic achievement (this national examination, which allows entry into university, has the advantage of being comparable for all students). Self-esteem was measured using Rosenberg's scale (RSE) [51]. Attitudes and beliefs towards cigarette companies derive from a translation into French of a pre-existing U.S. scale [37]. A high score corresponds to more negative attitudes or beliefs about cigarette companies. "Counterindustry" campaigns are also used in France, which makes attitudes and beliefs towards companies interesting to study. Tobacco and tobacco campaign perceptions scores are both *ad hoc*. A high score denotes more positive perceptions about tobacco itself and about the French tobacco prevention campaigns. The tobacco perception score used includes measures for reported predictors of smoking, such as outcome expectancies [34] (see table 1 for a description of the items of the scales).

### Statistical Methods

The analysis aims to identify the factors associated with cigarette use. The dependent variable is the number of cigarettes smoked per month. Modelling the number of cigarettes smoked per month makes it possible to model smoking status, as non-smokers are those smoking 0 cigarette per month.

The first step before conducting a statistical analysis is to adopt a strategy for handling missing variables. We were confronted with the problem because some students did not provide responses to some of the items in the questionnaire. Tobacco consumption, the dependent variable, is unknown for 10 students. In addition, if the 31 independent variables were considered together, in a complete case analysis only 490 students (67%) would be retained. We therefore used multiple imputation (MI) to avoid the loss of these students in the analyses [52]. Only the missing independent variables were imputed. Multiple imputation randomly draws observations from a fitted distribution for the covariates. For each imputed dataset, the missing data are filled in with values drawn randomly from this distribution. Analyses are performed on each data set as though the data had been completely observed. The analyses are then pooled to provide point and variance estimates for the effects of interest, using Rubin's rules [53]. Multiple imputation was performed using the Stata command "*ice*" [54].

The second task was to choose the statistical model to use for the analysis, taking into account the distributional characteristics of the dependent variable. The distribution of the number of cigarettes smoked was positively skewed (skewness coefficient of 2.7 in our sample, versus 0 had the data been normally distributed) and contained a large proportion of zero values (on account of non-smokers, representing 65% of our sample). This type of data can be modelled in a two-part model [55]. The mean quantity Q of cigarettes smoked by students is equal to the mean quantity Q^+ ^smoked by the students who are smokers, multiplied by their proportion π, since the (1-π) non smokers have a null consumption.



Two-part models use this property to split up the model into two independent elements. In the first part, the probability π of being a smoker is modelled. Logistic regression was used for this purpose; the sample used comprises all students. In the second part, the quantity Q^+ ^of cigarettes smoked by the smokers is modelled. A standard linear regression was performed on the log quantities for this part, estimated only on the subsample of smokers. The log-quantity of cigarettes smoked was used instead of the raw quantity so as to deal with skewness. Working on the log scale improves precision and robustness, but reverting to the original scale can be tricky. Duan's smearing estimator [56] was used as a means to return to the raw scale: it does not require normality of the error terms, but requires them to be homoscedastic.

All the final models were fitted using Stata. The standard errors were adjusted for the clustered nature of the data (as students are clustered within classes) using a cluster robust variance estimator [57,58].

Finally, once the model to be used for the analyses had been specified, the issue of identifying, among the 31 independent variables, those that were best suited to predicting cigarette consumption had to be dealt with. A classical set of methods for variable selection is based on stepwise regression. As these methods are based on multiple testing procedures, their results are questionable [59]. Stepwise methods are problematic in the presence of collinearity, and may include noise variables and select an unstable set of predictors [60].

A bootstrap method was therefore used for variable selection. It makes it possible to rank the strength of the association between each independent variable and tobacco consumption [61]. For each of the five imputed datasets, 200 bootstrap samples were generated and a backward variable selection was conducted on each. The stepwise selection was based on the AIC criterion, which avoids variable selection on the basis of hypothesis testing [62]. Variable selection using this bootstrap procedure was performed using R [63] and the "*stepAIC*" command.

How frequently a variable is retained after each of the 1000 stepwise selection procedures is an indicator of the strength of the association with the outcome. This procedure was applied independently in the two parts of the model.

Once the variables have been ranked according to their strength of association (i.e. the probability of being included in a model constructed using stepwise variable selection), only those above a certain threshold are selected. Inclusion thresholds for the variables to retain in the model vary [61]. In our case, an inclusion threshold of 70% would retain 12 and 10 covariates in parts one and two of the model, respectively. We favoured model parsimony (a model with fewer variables is simpler and reduces the risk of multicollinearity) and used an inclusion threshold of 80%. Part 1 of the model has 8 covariates and part 2 has 6.

The bootstrap selection procedure is only used to select which independent variables are to enter the model. Once a model is selected, the relative importance of each regression coefficient is then a measure of the effect of each predictor variable retained in the model.

## Results

### General characteristics of the sample

Females represented 79% of the students. Mean age was 22 years. In France, a student who has not repeated a year enters the second year of university at age 19–20. However, many nursing students have followed other studies before engaging in this profession, and they are therefore older (mean age of 25, versus 21 for the other students). While 61% of the students were under 21 years old, 5% were over 30 years old (one student was aged 64 at the last birthday). Smoking status according to selected student characteristics is presented in table 2.

**Table 2:** Student characteristics, smoking status and mean number of cigarettes smoked per day

**Characteristic**	**Non smoker**	**Former smoker**	**Occasional smoker**	**Daily smoker**
***Gender (p = 0.34)***				
• Male (21%)	56.77%	4.52%	19.35% (1.6)	19.35% (10.8)
• Female (79%)	57.80%	7.62%	19.86% (3.1)	14.72% (10.3)
***Academic discipline (p < 0.01)***				
• Sociology (36%)	52.14%	5.84%	19.84% (3.3)	22.18% (11.2)
• F. Language (12%)	72.94%	4.71%	14.12% (3.1)	8.24% (09.0)
• Medicine (19%)	75.74%	2.21%	17.65% (2.3)	4.41% (04.8)
• Nursing (34%)	47.97%	11.79%	22.76% (2.4)	17.48% (10.4)
***Age (p < 0.01)***				
• 18–19 (36%)	68.46%	3.85%	18.85% (2.6)	8.85% (11.0)
• 20 (25%)	60.44%	6.04%	17.03% (2.9)	16.48% (08.9)
• 21–23 (24%)	47.65%	4.71%	25.29% (2.8)	22.35% (10.7)
• 24–64 (15%)	42.59%	18.52%	18.52% (2.8)	20.37% (11.4)

Notes: Percentages in parentheses indicate the proportion of students in this category. Numbers in parentheses indicate mean number of cigarettes smoked per day for occasional and daily smokers. P-values are for the χ^2 ^test of independence between smoking status and student characteristics.

22% of the students were occasional cannabis smokers and 7% regular (i.e. smoking cannabis more than once a week). 60% were occasional alcohol drinkers and 9% regular (use at least 10 times in the last month). 22% were occasional binge drinkers and 7% weekly (at least 4 episodes in the previous month).

Only 16% of the students stated they had not been exposed to any tobacco prevention campaign in the month preceding the survey. The median number of campaigns reported for the others is 4.

### Tobacco use

Tobacco experimentation appears to be the norm in this sample, 73% of the students had tried a smoke a least once (the mean age of experiment was 14 years). Daily smoking (15% of the sample, usually smoking 10.41 cigarettes per day on average) and occasional smoking (20% of the sample, usually smoking 2.75 cigarettes per day on average) are less widespread. Former smokers represented 7% of the students.

Among smokers, only 4% usually never smoked cigarettes in packs while 64% never smoked hand-rolled cigarettes. 69% of hand-rolled cigarette smokers stated that the main reason for its use was that it was cheaper than cigarettes in packs.

It should be noted that few students (21% of occasional and 37% of daily smokers, 4% of former smokers and 1% of non smokers) stated they were against smoke-free universities. The idea of smoke-free universities is therefore widely accepted by students (overall, 76% supported the idea and 13% were indifferent).

### Ranking of the independent variables

The first part of the model was estimated on 721 of the 731 students for whom the quantity of cigarettes smoked was known and the second on 245 students, those who reported usually smoking at least one cigarette per month. The first part of the model predicts smoking status (i.e. smoker/non smoker) and estimates the probability of being a smoker, while the second predicts smoking intensity among smokers and estimates the number of cigarettes usually smoked in a month.

Table 3 gives the bootstrap-estimated inclusion probability (IP) of the 31 independent variables considered in each part of the model.

**Table 3:** Inclusion frequencies of the independent variables in each part of the model of tobacco use

	**Part 1: probability of smoking**		**Part 2: quantity smoked by smokers**	
**Variable**	**% retained**	**Rank**	**% retained**	**Rank**
Cannabis use	100.0	1	76.9	7
Position about smoke-free universities	99.8	2	90.6	2
Perceived friends' approval of regular smoking	97.7	3	90.2	3
Tobacco perception score	94.4	4	41.8	25
Perceived prevalence of smoking among friends	92.2	5	65.1	14
Frequency of being disturbed by people smoking in university	90.1	6	98.6	1
Binge drinking	86.7	7	49.6	22
Gender	84.3	8	53.9	20
Attitudes towards tobacco industry score	79.5	9	44.9	24
Knowledge of university tobacco policy	76.8	10	32.3	30
Parents together	74.5	11	46.2	23
Smoking prevalence in class	72.7	12	69.1	11
Perceived friends' approval of occasional smoking	66.4	13	54.8	18
Alcohol use	66.3	14	71.8	9
Number of friends	66.2	15	67.0	13
Cannabis use prevalence in class	54.0	16	75.7	8
BMI	53.2	17	36.6	28
Perceived smoking prevalence by peer students	51.6	18	70.2	10
Perceived exposure to tobacco prevention campaigns	51.4	19	60.0	16
Tobacco prevention campaigns perception score	50.8	20	67.4	12
Perceived friends' approval of smoking experimentation	48.4	21	55.9	17
Academic discipline	47.8	22	82.0	6
Binge drinking prevalence in class	41.0	23	39.2	27
Partner status (having one partner or not)	39.0	24	50.0	21
Number of friends in class	33.3	25	54.8	18
Age	30.7	26	83.9	4
Previous exposure to substance-use questionnaires	30.0	27	61.3	15
Final high school exam grade	29.7	28	35.1	29
Alcohol drinking prevalence in class	29.6	29	31.2	31
Self-esteem score	25.7	30	39.7	26
Beliefs about tobacco industry score	25.5	31	83.8	5

Social norms appear to be central predictors of the decision to smoke, as well as of the number of cigarettes smoked by smokers, although some measures are more salient than others. Perceived approval of regular smoking by friends ranks third in both parts of the model. Other injunctive norms such as perceived approval of occasional smoking and smoking experimentation have lower ranks (respectively 13^th ^and 21^st ^for part 1 and 18^th ^and 17^th ^for part 2 of the model). Descriptive social norms show less evidence of an association. Perceived prevalence of smoking among friends appears to be associated with smoking (ranking 5^th^) but not with the quantities smoked (ranking 14^th^). As for perceived prevalence of smoking among peer university students, it ranks 18^th ^for the model predicting smoking and 10^th ^for the one predicting the quantities smoked by smokers.

Other predictors that stand out are substance use (cannabis smoking and binge drinking), at least for the probability of smoking (ranking 1^st ^and 7^th ^respectively), and positions about tobacco itself and its acceptability in the university.

### A model predicting tobacco use

Table 4 presents the results of the model estimated using an inclusion probability threshold of 80% for the variables to retain in each part of the model. The first part models the probability of being a smoker using logistic regression. The exponentiated coefficients, corresponding to the odds-ratios (OR) of being a smoker, are therefore presented. The second part models the log-quantity of cigarettes smoked by the smokers. The effects of the coefficients on the raw scale are multiplicative, therefore the exponentiated coefficients are also presented.

**Table 4:** Two part model of the quantity of cigarettes smoked in a month

	Part 1: P(nb cig > 0) N = 721			Part 2: log(nb cig) N = 245		
Variable	OR	β/σ		exp(β)	β/σ	
Academic discipline (ref = sociology)						
• English as a foreign language				1.0318	0.09	
• Medicine	-			0.4828	-3.25	**
• Nursing				0.6809	-2.16	
Perceived approval of regular smoking by friends (ref = strong disapproval)						
• Approval	4.5358	5.08	***	2.9571	3.41	**
• Disapproval	1.6844	2.13	*	2.4925	6.28	***
Perceived proportion of friends smoking (ref = none)						
• <33%	1.2195	0.52				
• Half	3.4953	3.13	**	-		
• >66%	2.6591	2.61	**			
Smoke discomfort in university (ref = never)						
• Seldom	0.4227	-2.15	*	0.6747	-2.33	*
• Sometimes	0.2606	-3.18	**	0.2849	-4.45	**
• Often	0.1572	-3.19	**	0.1264	-4.29	**
Position about smoke-free universities (ref = against)						
• Indifferent	0.2288	-2.62	**	0.5718	-2.98	*
• Mostly for	0.1506	-3.99	***	0.5687	-1.99	
• Totally for	0.0756	-6.54	***	0.4794	-3.52	**
Tobacco perception score (high scores = positive perceptions)	1.1635	3.63	***	-		
Beliefs about tobacco industry score (high scores = negative beliefs)	-			1.0342	2.26	*
Binge drinking (ref = no)						
• Occasional (< 4 times/month)	2.9303	2.54	*	-		
• Weekly (≥ 4 times/month)	1.3186	0.39				
Cannabis use (ref = no use)						
• Occasional (≤ 1/week)	3.4140	3.62	***	-		
• Regular (> 1/week)	8.2666	2.13	*			
Gender (ref = male)	2.7103	2.80	**	-		
Age	-			1.0366	3.13	*
Constant (exponentiated)	0.2899	-1.40		30.9910	8.22	***
Duan's smearing estimator	Not applicable			2.2494	Not applicable	

*** p < 0.001; ** p < 0.010; * p < 0.050

Notes: Only independent variables with a bootstrap-estimated, backward selection inclusion probability ≥ 80% were included in each part of the model; - = variable not included; OR = odds ratio; β/SE = Wald test statistic (β = regression coefficient, σ = standard error of the coefficient). Part 1 models the probability of being a smoker using logistic regression, part 2 models the log-number of cigarettes smoked in a month by smokers using linear regression.

The approval of regular smoking by friends is used as an example. Part 1 models the probability of smoking. The OR of being a smoker is 4.54 for students reporting that their friends approve of regular smoking compared to those whose friends strongly disapprove of it. Part 2 models the number of cigarettes smoked in a 30-day month by smokers. Smokers reporting that their friends approve of regular smoking smoke 3 times more than those reporting that their friends strongly disapprove of it.

Perceived approval of regular smoking by friends is therefore associated with an increased risk of being a smoker and with an increased number of cigarettes smoked among smokers.

More generally, the risk of smoking increases with cannabis use, low approval of smoke-free universities, perceived approval of regular smoking by friends, positive perceptions of tobacco, high perceived prevalence of smoking among friends, low levels of discomfort from smoking in the university, occasional binge drinking, and being a female student.

The quantity smoked by smokers increases with low levels of discomfort from smoking in the university, low approval of smoke-free universities, perceived approval of regular smoking by friends, age, negative beliefs about tobacco industry practices, and being a sociology student.

### Sensitivity analyses

A sensitivity analysis was performed using a linear regression on the ranks of the quantities of cigarettes smoked by smokers to check the adequacy of the model, given the asymmetric distribution of the raw data. The results were similar (direction of the association and statistical significance) to those obtained using the log-quantity of cigarettes smoked.

The analyses presented here include a small proportion of students (5%) aged over 30. These students are also eligible for prevention purposes, and were therefore retained in the sample. In order to assess the impact of this decision on the results, we also performed the analysis excluding students aged over 30. In this restricted sample, the variables selected at the 80% bootstrap selection threshold are broadly similar, except, in the first part of the model (probability of tobacco use), for binge drinking (86.7% probability of inclusion in the baseline model and 78.6% in the restricted sample model), for the attitudes towards the tobacco industry score (probabilities of inclusion of 79.5% vs. 85.8%) and for family structure (probabilities of inclusion of 74.5% vs. 81.7%). Among students aged 30 years or under, the model coefficients are very similar to those reported for all students. While family structure fails to reach statistical significance (p = 0.18, Wald test), attitude towards the tobacco industry is significant (p = 0.02). In this alternative model (estimates not shown), negative attitudes towards the tobacco industry reduce the probability of being a smoker.

### Gender and academic differences in tobacco use

In the model retained (see table 4), females are more likely to be smokers than males. While no association was found between smoking and gender in a non adjusted analysis (33% of females vs. 38% of males were smokers, OR = 0.81, p = 0.31, chi-squared test), this is attributable to a Simpson's paradox [64], a situation were an association between two variables is inverted when the population is partitioned – and here students are partitioned by cannabis use. Female cannabis users and female non-cannabis users were both more frequently tobacco users than males (73% vs. 63% and 21% vs. 15%). We thus identify an association between gender and smoking only after adjusting for cannabis use (OR = 1.68, p = 0.03, Wald test in a logistic regression model containing gender and cannabis use as predictors). The association remains in the same direction after excluding nursing students from the sample (nursing students are often female and, in France, have a high prevalence for smoking), although it is not statistically significant on account of the reduced sample size (OR = 1.62, p = 0.09).

Among smokers, according to the model retained (see table 4), medical students smoke on average 48% of the amount of cigarettes smoked by sociology students. The difference between nursing students and sociology students is not significant. The difference observed for medical students can be explained by the fact that, while the proportions of occasional and daily smokers are balanced for sociology students (20% and 22% respectively), most smokers in medicine are occasional smokers (18% of medical students are occasional smokers and 4% are daily smokers).

## Discussion

The aim of this study was to assess the relative importance of social norms, when compared to other potential predictors (e.g. other substance use...), in explaining cigarette consumption in French university students. We will now review how social norms fare as predictors of cigarette use, what other predictors are also identified, and what the policy implications of these findings are.

### The role of social norms factors

It appears that social norms are indeed associated with cigarette use. Proximal peers (friends) are, however, more influential than more distal peers (students). We will first review the norms identified in this analysis, then focus on possible explanations for the findings.

#### Proximal norms: the role of friends

Proximal peer norms can be injunctive or descriptive. Perceived prevalence of smoking among friends, a descriptive norm, was only retained in the model predicting smoking. Two mechanisms could explain this association: peer influence (the student is influenced to smoke by his friends) or peer selection (he/she selects friends by their smoking status). Longitudinal studies among young adolescents show that both influences can explain tobacco smoking [65]. Although the present study does not enable the estimation of actual prevalence of smoking among the friends, this variable has been shown to be a less important predictor than perceived prevalence in other studies [2,6]. Injunctive norms are more influential. Perceived approval of regular smoking by friends is the social norm measure that is the most likely to be associated with smoking, and it is the only social norm retained in relation to smoking quantities. In alcohol research, students have been found to overestimate injunctive norms more than descriptive norms [66]. Since injunctive norms are subject to greater degrees of misperception, it is not surprising that these norms are also more associated with tobacco use. A study among high school students entering university reported that believing peers approved of smoking was a predictor of progression from smoking experimentation to higher levels of smoking [35].

#### Distal norms: the role of peer students

Contrary to proximal peer norms, the more distal peer norms appear to have a limited role in tobacco consumption. Perceived smoking prevalence among peer students was not included in the model as its bootstrap-estimated inclusion probabilities (51.6% in part 1 and 70.2% in part 2 of the model) were below the selection threshold retained. Still, this descriptive norm has been identified in other studies as a correlate of smoking. In a population of students from historically black colleges and universities in the USA, overestimation of smoking prevalence was found to increase the risk of smoking [67]. Moreover, at Virginia Commonwealth University, a campaign based on the message that "7 of 10 college students don't smoke" was successful in changing perceptions of peer smoking prevalence and in controlling the evolution of the mean number of cigarettes smoked per month [68]. The associations observed in U.S. higher education students might not hold for French students, or could be masked by the other, stronger associations (norms regarding friends) measured in our study.

#### A model explaining the association between norms and tobacco use

Our study does not try to explain why the association identified between norms and cigarette use exists. Some models have been proposed, such as the theory of normative social behaviour [69]. According to this theory, descriptive norms affect behaviours through interactions with injunctive norms, outcome expectations and group identity. The authors later extended this model, adding peer communication as a moderator in the relationship between descriptive norms and behaviours [70]. This extension was tested in the domain of alcohol use, but it could explain why, in our study, norms related to friends are more influential than norms related to peer students. As we do not measure group identity in this study, we cannot test its influence on the relationship between descriptive norms and smoking behaviours.

### Other factors associated with smoking

Social norms are not the only variables for which an association with cigarette use is identified. Other substance use, sociodemographic characteristics, and perceptions about tobacco, its use and its production are also retained in our analysis.

#### Other substance use

Cannabis and, to a lesser extent, binge drinking, are associated with smoking, but are not retained as predictors of the quantities smoked by the smokers. These behaviours are health-threatening and unhealthy behaviours are associated with each other [71]. Furthermore, cannabis in France is mostly smoked in joints with tobacco. The association between tobacco and alcohol and other substance use is also reported among U.S. university students [16,24], and alcohol has been thought to play the role of a complement to smoking for "social smokers" [72].

One alternative explanation for these associations would be that tobacco is a gateway drug for other drug use, as has been explored among U.S. high school students [73]. However, in a study among college students who had not smoked previously, alcohol use was associated with a higher likelihood of smoking initiation, reversing the direction of the association [9]. The temporal relationship between tobacco and alcohol use in the U.S. might not be found in countries such as France where alcohol is culturally initiated at younger ages (the legal age for buying alcohol is of 16).

#### Sociodemographic characteristics

Females are more likely to smoke cigarettes than males, and age increases the quantity of cigarettes smoked by smokers. Because of the cross-sectional nature of this study, we cannot know if this is a generation effect (previous generations smoked more) or an age effect (as smokers become addicted, they increase their consumption).

The academic discipline is also associated with the number of cigarettes smoked by smokers. Smoking sociology students are the group with the highest consumption, while medical students are those with the lowest consumption. Medical students could be one of the groups the most aware of the negative consequences of smoking and this knowledge might prevent heavy consumption levels. However, nursing students are also trained about the health consequences of smoking, but sociology and nursing students who smoke do not differ in the quantities of cigarettes smoked.

#### Perceptions related to tobacco

A positive perception of tobacco was associated with an increased risk of being a smoker and was not included as a predictor of the quantities smoked by smokers.

Conversely, beliefs about tobacco industry practices are associated with quantities smoked by smokers. Surprisingly, negative beliefs about the tobacco industry are associated with higher consumption. Perhaps heavy tobacco users are more addicted and more willing to blame tobacco industry for it.

The position about smoke-free universities is also a predictor of smoking. Not being favourable to smoke-free universities is associated with an increased risk of smoking and higher consumption, as has been observed in the U.S. [33]. Our study extends this finding by showing that being supportive of smoke-free universities is not only negatively linked to smoking but also negatively linked to the quantities smoked by smokers. This variable, like the frequency of reporting smoke discomfort in university, also negatively associated with being a smoker and with quantities smoked by smokers, is more probably a consequence of tobacco use than a cause of it.

### Policy implications

The results of this study have many implications for prevention policies. They result from the methodological choices made. First, as multiple predictors have been considered in addition to social norms, the relative importance of social norms can be assessed.

#### Non-social norms variables of interest for prevention

At the local, French level, we provide evidence of a strong demand among students for public health measures against tobacco, as shown by the fact that even smokers are for a majority supportive of smoke-free universities. Prevention campaigns are always subject to psychological reactance effects, in which individuals feeling their freedom is threatened will react by doing the opposite of what is advocated by the campaign [74]. The positive attitude towards tobacco-use prevention measures observed in this survey indicates that such reactance effects are less likely, and provides a favourable ground for university-based prevention actions.

Furthermore, the association found between tobacco and other substance use (cannabis and binge drinking) highlights the need to elaborate prevention campaigns that are not limited to tobacco use alone.

#### Social norms factors

More importantly, we find evidence that social norms play an important role as predictors of cigarette consumption. The role of social norms has been evidenced in the U.S. for younger populations or other substance use (at university level, mostly alcohol). We show that social norms concerning tobacco also play a role in other populations (French university students). This finding implies that prevention campaigns based on social norms should be tested at university level to reduce tobacco use in these populations. However, perceived prevalence of smoking among peer students, a descriptive norm often used in social norm interventions, is not retained in our model. Instead, more proximal peers (our study measured friend-related norms, but partner-related norms have been documented to be even more predictive of smoking [75]) and injunctive norms appear to be more predictive of smoking. It is possible to use injunctive norms in prevention interventions (e.g. "75% of students disapprove of smoking"), and this possibility should be tested for tobacco prevention. Since friend-related (proximal peer) norms are more influential on students than peer student norms, prevention interventions in campuses could be expected to be more effective if students elect their friends among students from the same campus. In any case, many authors recommend that smokers should be considered not only as individuals but as members of a social group [70,76,77]. Another strategy would be to reinforce campus group identity by demonstrating similarity with other students [78], which could be achieved through correction of misperceived norms.

#### Implications deriving from the model used

A second set of implications, perhaps more speculative, derives from the fact that our two-part analysis predicts smoking status and smoking quantities among smokers separately. This is potentially of interest for harm-reduction strategies.

A potential harm-reduction approach would be to advise smokers who do not want or are unable to quit smoking to reduce their use. This strategy would prove effective if it does not encourage non-smokers to initiate smoking and if reducing the quantity of cigarettes smoked does reduce the risk of negative health consequences. The use of two-part models could prove useful in planning prevention interventions including a harm-reduction campaign, as it enables separate consideration of which factors are likely to impact tobacco initiation (the probability of smoking) and which are likely to impact the quantities of cigarettes smoked. Common factors, such as perceived approval of smoking by friends, could be given a priority, since they have the potential to reduce both the proportion of smokers and the quantities smoked by smokers.

### Limitations

There are three aspects of the study design to be taken into account. First, our study did not attempt to describe all academic disciplines, as it was restricted to 4. Since the aim of the study was not to describe substance use epidemiology among university students but to evaluate the potential impact of a social norm prevention approach on substance use reduction, an exhaustive sample of academic disciplines was not an objective.

Second, the conclusions of this study are based on a cross-sectional sample. This design only allows for measures of association, and we do not know whether the independent variables occur before or after the dependent variable in the model.

Finally, the self-reported nature of the data also needs to be taken into account. There are two causes of inadequate validity of self-reported behaviours: cognitive factors (related to internal processing of the questions, potentially subject to recall errors) and situational factors (related to the external environment, potentially subject to social desirability biases). The validity of the responses will therefore depend on the responder's perception of the social disapproval of tobacco use. As the questionnaire was presented as confidential and anonymous, and since smoking is not illegal at the ages considered in this study, the probability of a response bias is minimized. In fact, studies show that self-reported behaviours related to tobacco are accurate when a self-administered questionnaire is used, and there is strong agreement between self-reported and biochemical measures of tobacco use [79,80].

Another factor to be taken into account is the fact that students that missed class at the time of the survey did not complete the questionnaire. This could have an impact on prevalence estimations if those missing differed from the students present in class (if smokers were more likely to miss class, for instance).

## Conclusion

Tobacco use predictors have been extensively studied, mostly among adolescents. We present here results for an older population (university students), using a methodology that makes it possible to appraise an extensive number of predictors for smoking and quantities smoked by smokers. We show that the factors associated with being a smoker are not necessarily those associated with the quantities smoked by smokers. Among the predictors identified, two categories are of particular interest: other substance use, which appears to be associated with smoking, and social norm measures, both injunctive and descriptive, associated both with smoking and with quantities smoked by smokers.

Although we find an association between certain descriptive norms and smoking, some measures appear to be more promising than others. An intervention that only corrects misperceived tobacco use prevalence in a class is unlikely to be effective, as there is only weak evidence of an association between the perceived prevalence of smoking and being a smoker [7]. Conversely, an intervention changing the perceived approval of smoking by friends or the perceived prevalence of smoking by friends (on the basis that most friends are from the same class) can be expected to be more effective. Still, the presence of an association between a factor and a risky behaviour does not necessarily imply that modifying this risk factor will have an impact on the behaviour. Further research is needed in order to conclude on the effectiveness of social norm interventions to reduce tobacco smoking among French higher education students.

## Competing interests

The authors declare that they have no competing interests.

## Authors' contributions

LRF, BD and MR designed the study and wrote the protocol. LRF and MR undertook the pilot study. LRF and BD participated in the collection of the data. BF validated the statistical analysis plan. LRF managed the literature searches and summaries of previous related work, undertook the statistical analysis, and wrote the first draft of the manuscript. All authors contributed to and have approved the final manuscript.
