# Are social norms associated with smoking in French university students? A survey report on smoking correlates

**DOI:** 10.1186/1747-597X-4-9

**Published:** 2009-05-05

**Authors:** Lionel Riou França, Bertrand Dautzenberg, Bruno Falissard, Michel Reynaud

**Affiliations:** 1 INSERM U669, Maison de Solenn-97, Bvd de Port-Royal, 75679 Paris Cedex 14, France; 2 Université Paris 6, Pierre et Marie Curie, 4 place Jussieu, 75005 Paris, France; 3 Faculté de Médecine Pierre et Marie Curie, UPRES EA2397-91, 105 Bvd de, l'Hôpital, 75013 Paris, France; 4 Assistance Publique-Hôpitaux de Paris, Service de Pneumologie et, Reanimation, GH Pitié Salpêtrière Division Montyon, 75651 Paris Cedex 13, France; 5 Université Paris-Sud 11 and Université Paris Descartes 5, UMR-S0669, Maison de, Solenn, 97 Bvd de Port-Royal, 75679 Paris Cedex 14, France; 6 Assistance Publique-Hôpitaux de Paris, Hôpital Paul Brousse, Département, de Santé Publique, 12 Ave Paul-Vaillant-Couturier, 94804 Villejuif Cedex, France; 7 Assistance Publique-Hôpitaux de Paris, Hôpital Paul Brousse, Unité, Fonctionnelle d'Addictologie, 12 Ave Paul-Vaillant-Couturier, 94804 Villejuif, Cedex, France

## Correction

Following the publication of the article [1], we noticed that the Competing interests section was incomplete and should read as follows:

BD is President of ACTIF (*Alliance Contre le Tabac en Île-de-France*), a non-profit association which promotes smoking prevention and provided funding for this study. BD's involvement with the ACTIF is on a voluntary basis and he does not receive any financial compensation for his work. He was responsible for decisions to partially fund this study.

## Competing interests

The authors declare that they have no competing interests.
